# Morphological Changes in the Suprachiasmatic Nucleus of Aging Female Marmosets (*Callithrix jacchus*)

**DOI:** 10.1155/2014/243825

**Published:** 2014-06-02

**Authors:** Rovena Clara G. J. Engelberth, Kayo Diogenes de A. Silva, Carolina V. de M. Azevedo, Elaine Cristina Gavioli, Jose Ronaldo dos Santos, Joacil Germano Soares, Expedito S. Nascimento Junior, Judney C. Cavalcante, Miriam Stela M. O. Costa, Jeferson S. Cavalcante

**Affiliations:** ^1^Laboratory of Neurochemical Studies, Physiology Department, Biosciences Center, Federal University of Rio Grande do Norte, Natal, RN, Brazil; ^2^Laboratory of Chronobiology, Physiology Department, Biosciences Center, Federal University of Rio Grande do Norte, Natal, RN, Brazil; ^3^Behavioral Pharmacology Laboratory, Department of Biophysics and Pharmacology, Federal University of Rio Grande do Norte, Natal, RN, Brazil; ^4^Biology Department, Federal University of Sergipe, Aracaju, SE, Brazil; ^5^Laboratory of Neuroanatomy, Morphology Department, Biosciences Center, Federal University of Rio Grande do Norte, Natal, RN, Brazil

## Abstract

The suprachiasmatic nuclei (SCN) are pointed to as the mammals central circadian pacemaker. Aged animals show internal time disruption possibly caused by morphological and neurochemical changes in SCN components. Some studies reported changes of neuronal cells and neuroglia in the SCN of rats and nonhuman primates during aging. The effects of senescence on morphological aspects in SCN are important for understanding some alterations in biological rhythms expression. Therefore, our aim was to perform a comparative study of the morphological aspects of SCN in adult and aged female marmoset. Morphometric analysis of SCN was performed using Nissl staining, NeuN-IR, GFAP-IR, and CB-IR. A significant decrease in the SCN cells staining with Nissl, NeuN, and CB were observed in aged female marmosets compared to adults, while a significant increase in glial cells was found in aged marmosets, thus suggesting compensatory process due to neuronal loss evoked by aging.

## 1. Introduction

The suprachiasmatic nuclei (SCN) are a pair of neurons located in the anterior hypothalamus above the optic chiasma, [1, 2] and it is constituted of about 8,000 to 10,000 neurons [3–5]. Since the 1970's, the SCN has been considered the central pacemaker [6, 7] that controls physiological and behavioral rhythmic oscillations in mammals [8]. Approximately 10% of SCN neurons express VIP in the core subdivision, while around 20% (which means about 2,100 neurons) of SCN in the shell part express VP [9]. Other neurotransmitters, besides VIP and VP, have been described in terminals and perikarya in the SCN, such as neuropeptide Y [10–12], 5-HT [13] and calbindin (CB) [10].

In humans, many factors attempt to explain the aging process; among them, mitochondrial dysfunction [14] and oxidative stress [15] appear to be strong candidates able to promote alterations in aged organisms. Aging process alters structural complexity of the central nervous system, resulting in neurotransmitters alterations [16], atrophy of total gray matter [17], and soma size and dendrites [18].

During aging, changes in morphological, neurochemical, and circadian rhythms can be observed [19]. The effects of aging on circadian rhythm range from simple alterations in physiological functions to impairment of cognitive performance [20]. However, neonatal SCN tissue transplantation in old hamsters increased life span and restored normal rhythms in many physiological mechanisms [21]. Thus, it supports that longevity appears to be correlated with regulated circadian rhythm activity.

In mammals, changes in basic parameters of circadian rhythms have been showed, such as alterations in length period, besides reduction of amplitude and phase duration [22, 23]. These alterations in circadian rhythms can be caused by changes in neurochemical and morphological aspects during aging. Some studies have reported contradictory results with regard to the decrease in neuronal cells in the SCN in rodents and primates [24–26]. In female aged rodents, there were no reported alterations in the SCN neuron numbers, whereas Roberts et al. [25] showed reductions in SCN neurons in aged female Rhesus monkey. Besides,* post mortem* studies in human beings showed that neurodegenerative processes occur in the SCN during senescence, suggesting progressive deterioration of the circadian pacemaker [27, 28].

Considering that neuronal nuclear protein (NeuN) has been widely used as a neuronal marker, while, in marmosets, CB is used as an important cell marker of the SCN [10, 29], in the present study we aimed to assess in the* Callithrix jacchus* a new world primate, the number of neurons in the SCN in aged female. To this aim, a morphometric analysis using Nissl staining and the immunoreactivity of SCN cells to NeuN, CB, and glial fibrillary acidic protein (GFAP—the main intermediate filament protein in mature astrocytes [30]) were performed in adult and aged female animals.

## 2. Material and Methods

### 2.1. Experimental Animals

Four young adult female marmosets and four aged female marmosets (see Table 1 for animals details) from the Primatology Center of the Federal University Rio Grande do Norte, Natal, Brazil, were used in this study. The use of the animals was approved by the Brazilian Environmental Protection Agency (IBAMA Register number 1/24/92/0039-00). The animals were kept in cages measuring 2,00 × 1,00 × 2,00 m and housed under natural conditions of temperature and humidity in a light-dark cycle, with food and water being freely available. The experimental procedures were in accordance with the Brazilian law number 11.794/2008 for animal experimental use. All experiments were approved by the local ethic committee for animal use (CEUA-UFRN number 026/2010).

### 2.2. Anaesthesia and Perfusion

The procedure perfusions in all animals were performed between 9–11 am. The animals were given tramadol hydrochloride and xylazine as preanaesthetic medication, both at the dose of 5 mg/kg intramuscularly and maintained on inhalation anaesthesia with isoflurane and 100% oxygen administered by a mask. After the administration of the anaesthetic drugs, animals were perfused transcardially with 400 mL phosphate-buffered saline (PBS), pH 7.4, containing 500 IU heparin (Liquemin, Roche, São Paulo, Brazil), followed by 700 mL 4% paraformaldehyde in 0.1 M phosphate buffer, pH 7.4. Brains were removed from the skull, postfixed in the same fixative solution for 2–4 h, and transferred to a solution containing 30% sucrose in 0.1 M PBS, pH 7.4. Each brain was serially cut in the coronal plane into 30 mm thick sections with a freezing sliding microtome. The sections were placed sequentially in six compartments (one section per compartment), with the distance between one section and the next in the same compartment being approximately 180 *μ*m. All sections in the six compartments were stored in antifreezing solution. Each compartment was then individually processed to reveal one of the following substances: NeuN, CB, and GFAP. In all cases, one of the compartments was processed by the Nissl technique with thionin dye (for technique details see [31]).

### 2.3. Tissue Processing and Data Acquisition

For immunostaining, free-floating sections of the one compartment were washed in phosphate buffer and incubated with the primary antibodies to NeuN (mouse anti-NeuN, Chemicon Internat. Inc., Temecula, CA, USA, Lot number LV1457494, dilution 1 : 1000), CB (mouse anti-CB, Sigma-Aldrich, Saint Louis, MO, USA, Lot number 031M4859, dilution 1 : 1000), and GFAP (mouse anti-GFAP, Sigma-Aldrich, Sain Louis, MO, USA, lot number 037K4759, dilution 1 : 1000) for 18–24 h at room temperature, containing 2% normal donkey serum in 0.3% Triton X-100 and 0.1 M phosphate buffer, pH 7.4. Next, the sections were incubated with the biotinylated secondary antibody (rabbit anti-mouse IgG; Sigma-Aldrich, Saint Louis, MO, USA, Lot number 072K4876, dilution 1 : 1000), for 90 min, followed by incubation with the avidin-biotin peroxidase solution (ABC Elite kit, Vector Labs, Burlingame, CA, USA) for 90 min. The reaction was developed by the addition of diaminobenzidine tetrahydrochloride (Sigma, St. Louis, MO, USA) and 0.01% H_2_O_2_ in 0.1 M phosphate buffer, pH 7.4. The sections were washed (5x, 5 min) with 0.1 M phosphate buffer, pH 7.4, between each step and at the end of the procedure. The sections were then dried, dehydrated in a graded alcohol series, cleared in xylene, and coverslipped with neutral mounting medium (DPX; Sigma). Sections in which the primary antibodies were omitted and replaced with normal serum from the same species served as controls. Under these conditions, staining was completely abolished. All the immunostainings, for specific substance, were performed concomitantly, minimizing possible differences in background between the animals.

The sections were examined under bright field illumination on a light microscope (Olympus BX-41) and images were captured using a CCD camera (Nikon DXM-1200).

### 2.4. Morphometric Analysis

In order to estimate the number of SCN cells, six images (objective 40x) of each animal, aged and adult, were selected: one at the rostral level, three at medium level, and two at caudal level, representative of the rostrocaudal extension of area of interest. For all solutions used as cell markers, we use only the right side of each hemisphere to the cell count. A rectangle measuring 125 um × 100 um, corresponding to 52% of the total area of the SCN, was extracted of every image and the number of cells found in this area was counted. All images (adults and aged) were randomly renamed so that the counter did not know which animal belonged to the image, featuring a blind count. Thus, it minimized the suggestive effects of knowing the sample. Some histological characteristics presented by neurons in the thionine dye were used as selection criteria in the cell counting, for example, rounded shape, strong staining, and evident nucleolus.

Additionally, GFAP levels in the SCN were assessed by analyzing relative optical densitometry (ROD) of images using the software Image J (Version 1.46i, NIH). Six representative sections of the rostrocaudal extension of SCN were chosen. In each section, fields bypassing throughout the SCN were analyzed. The medium pixels in the target area were subtracted from de medium values of a control region (areas that should not have specific GFAP staining) of the same tissue (optic chiasma). Finally, all values were normalized considering the control group, in order to evaluate proportional alterations.

### 2.5. Data Analysis

To confirm whether there are differences between the animal groups, the nonparametric Mann-Whitney test was applied. Correlations between age of animals and morphometric values were performed using the Spearman test (*r*). The level of significance was set at *P* < 0.05.

## 3. Results

**Figure 1:**
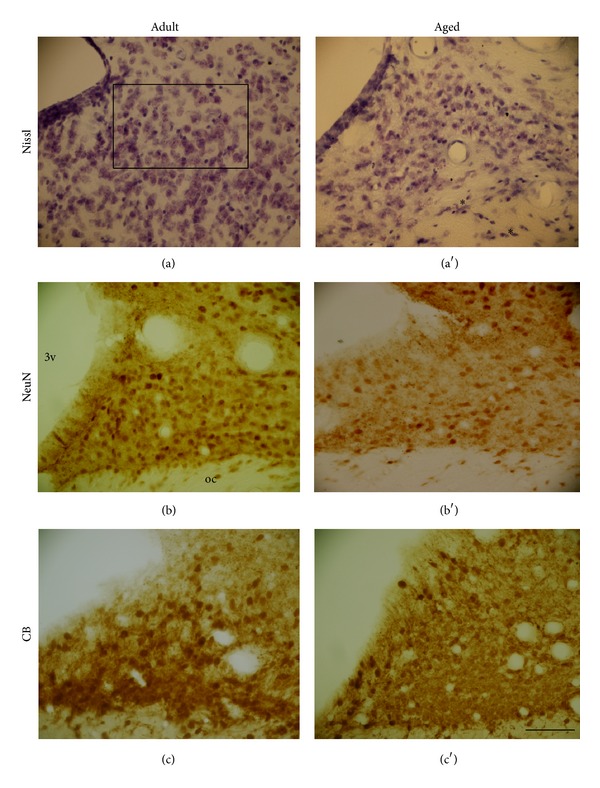
The suprachiasmatic nucleus. Digital images of coronal sections at hypothalamic level of the marmoset brain showing SCN at Nissl stained sections in adult (a) and aged (a′) animals; ((b) and (b′)) NeuN-immunostained; and ((c) and (c′)) CB-immunostained. Asterisk (*) shows degenerative areas and the rectangle in the figure represents the count area. CB: Calbindin; 3v: third ventricle; oc: optic chiasm. Scale bar: 50 *μ*m.

In SCN sections Nissl-stained (Figures 1(a) and 1(a′)), or marked to NeuN-IR (Figures 1(b) and 1(b′)) and CB-IR (Figures 1(c) and 1(c′)), a decrease in the number of cells was observed in aged marmosets when compared with adult animals. In aged animals, sections stained with Nissl (Figure 1(a′)) and NeuN (Figure 1(b′)) revealed many whitish areas throughout the SCN. However, these whitish areas were not observed in adult animals, which suggest the occurrence of neurodegeneration (Figures 1(a) and 1(b)). For immunostaining to CB, it is also possible to qualitatively observe a decrease in the number of cells available in the SCN of aged marmosets (Figure 1(c′)) compared to adult animals (Figure 1(c)).

**Figure 2:**
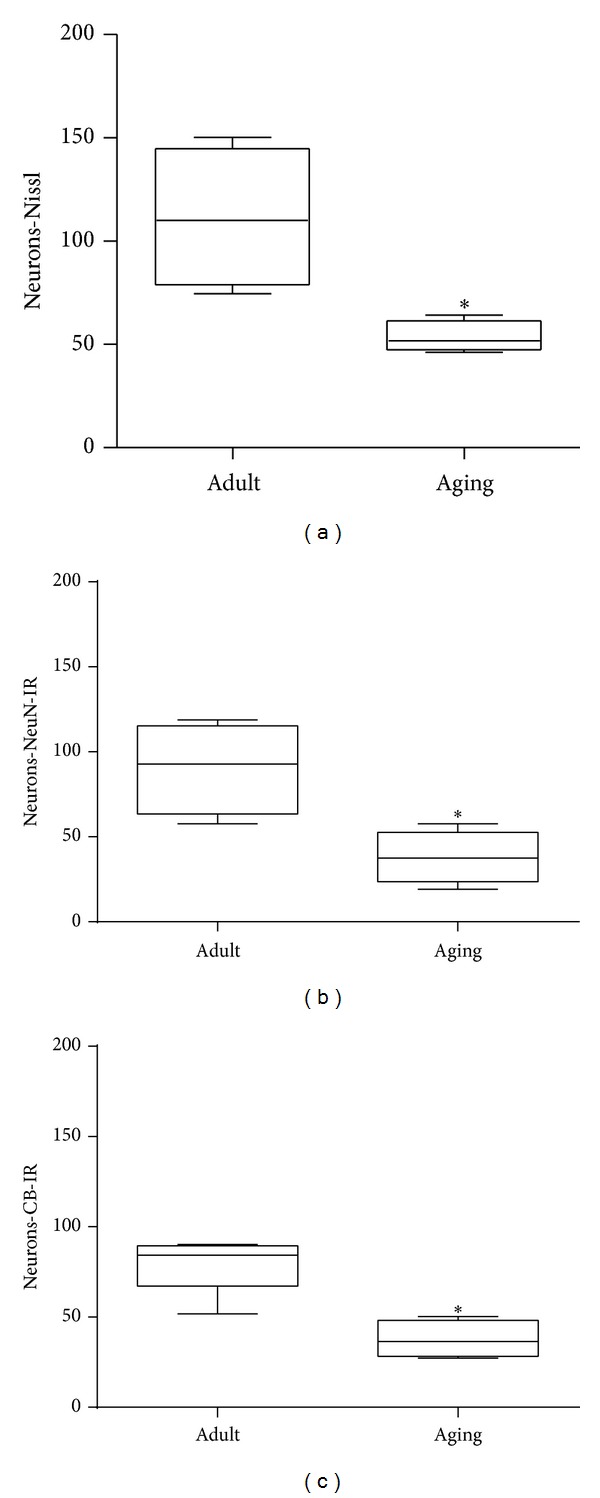
Effects of age on the number of neurons in the suprachiasmatic nucleus (SCN) of the marmoset* Callithrix jacchus:* (a) number of neurons for Nissl, (b) NeuN, and (c) CB-IR (Calbindin immunoreactivity). Mann-Whitney test revealed statistically significant difference between groups (Adult and Aged) for all stained. Data are expressed as median and interquartile intervals (q3–q1). *P < 0.05 compared to adult group.

These qualitative decreases in the number of cells in SCN sections observed in photomicrographs (Figure 1(c′) versus Figure 1(c)) were quantitatively documented. Mann-Whitney test revealed a significant decrease in the number of cells in the SCN stained by Nissl technique in aged compared to adult marmosets (adult marmoset: 110.0 (133.37–87.81); aged marmoset: 51.8 (55.62–49.64); data are represented as median (interquartile range q3–q1); *P* = 0.0209; Figure 2(a)) and NeuN (adult marmoset: 92.8 (108.06–75.12); aged marmoset: 37.4 (42.68–32.5); data are represented as median (interquartile ranges q3–q1); *P* = 0.0294; Figure 2(b)).

**Figure 3:**
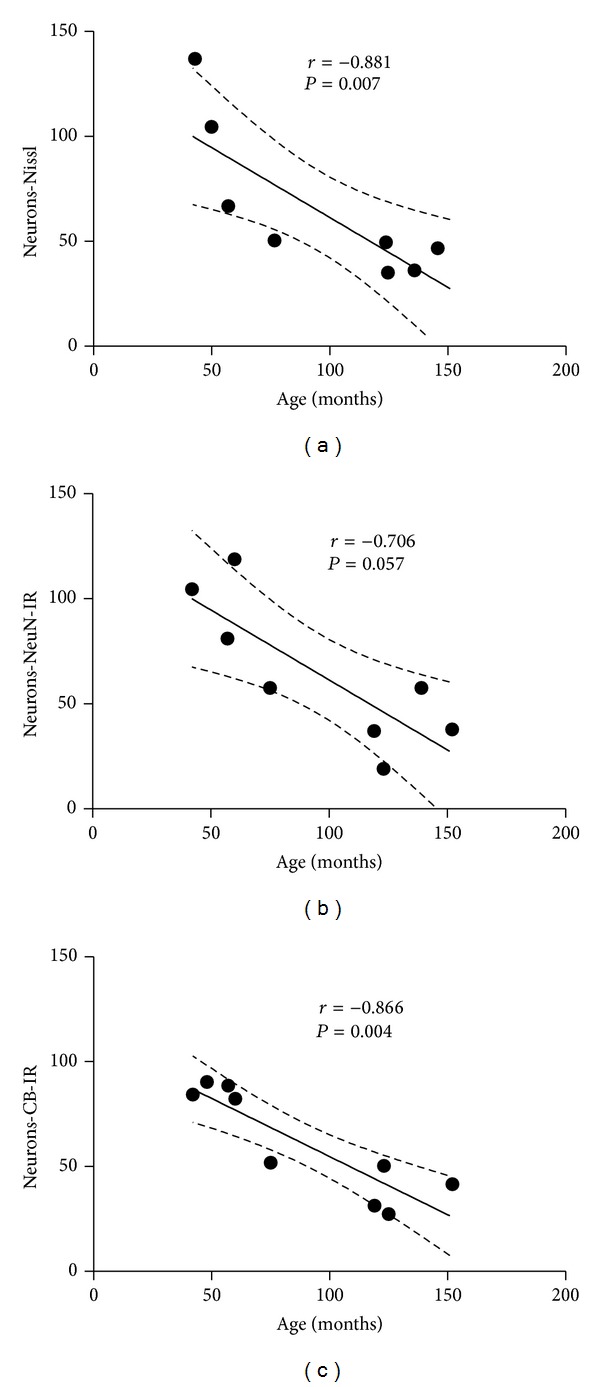
Correlation between cellular count and age in marmoset (*Calllithrix jacchus*) suprachiasmatic nucleus (SCN). Significant correlations (Spearman correlation—*R*) when *P < 0.05.

Mann-Whitney test showed a significant decrease in the number of neurons CB-stained in the SCN of aged female marmoset compared to adult female marmosets (adult marmoset: 84.3 (85.31–74.62); aged marmoset: 36.4 (43.68–30.25); data are represented as median (interquartile ranges q3–q1); *P* = 0.0143; Figure 2(c)). Using Spearman test, a significant negative correlation between age and counting of Nissl-stained and CB-IR in SCN neuronal cells was found, respectively (*r* = −0.881, *P* = 0.007, Figure 3(a); *r* = −0.866, *P* = 0.004, Figure 3(c)). Additionally, a marginal negative correlation between age and counting of NeuN-IR SCN cells was observed (*r* = −0.706, *P* = 0.057, Figure 3(b)).

**Figure 4:**
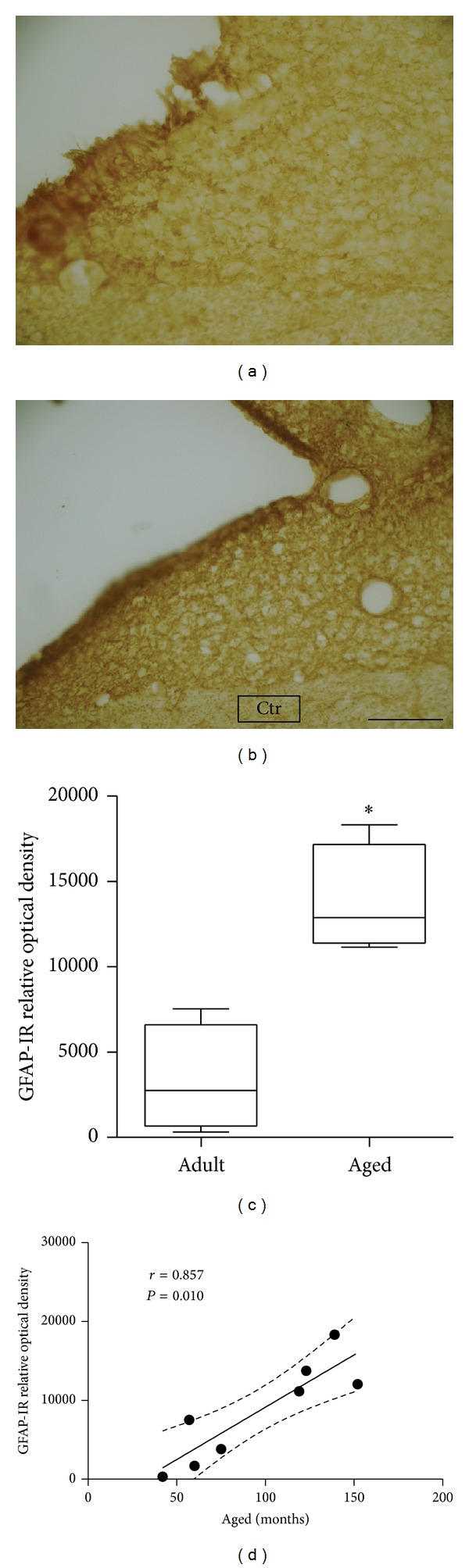
GFAP expression in SCN. Digital image of coronal section showing GFAP expression in adult SCN (a) and aged (b). Relative optical density measured showed a significant increase of GFAP expression in aged SCN compared to adult marmoset (c). Besides, Spearman test pointed to a significant positive correlation between GFAP-IR and age. Significant correlations (Spearman correlation—*R*) when *P < 0.05. ctr: control area. Scale bar: 50 *μ*m.

Considering GFAP immunoreactivity in the SCN, Mann-Whitney test revealed a significant increase in GFAP expression in aged marmosets compared with adult animals (adult marmoset: 2745.8 (4737.7–1339.1); aged marmoset: 12880.1 (14877.3–11807.3; *P* = 0.0209; Figure 4(b)). A significant positive correlation between age and GFAP immunoreactivity expression was found (*r* = 0.857; *P* = 0.010; Figure 4(c)).

## 4. Discussion

NeuN immunoreactivity has become an excellent marker for neuronal phenotypes that along with the traditional Nissl technique provides a new framework to structural and morphological studies [32], including the SCN [33]. The expression of NeuN has been shown in studies which verify neuronal death associated to age [34], since it has been proposed that immunoreactivity to NeuN depends on the phosphorylation state of protein [35], and aging could alter levels of cellular protein phosphorylation, resulting in neuronal loss [36].

Some papers reported that senescence influences the brain volume, showing that some regions are more susceptible to age-related plasticity than others [37, 38]. Regarding SCN, stereological studies have shown a decrease in the number of neurons in the SCN in humans with neurodegenerative diseases, such as Alzheimer's, and in aged people [39, 40]. A study in aged rodents of both gender without any neurodegenerative disease showed a decrease in the number of SCN cells, with a significant reduction of total neuronal volume [26]. A recent study with nonhuman primates (rhesus monkey) showed neurodegeneration in adult males and aged females [25]. Aged marmosets that served as the sample for cell counting in our results were all females with advanced ages (10–13 years) within the period of reproductive senescence [41, 42]. These aged marmosets showed a significant neuronal loss in the SCN, in which cell counting was performed by Nissl staining procedure and NeuN immunoreactivity. Similar results were reported in other studies using aged females rat and rhesus monkeys [25, 26].

Studies have suggested a possible role for progesterone in protecting neurons against neurodegeneration mainly during the senescent period in which hormone levels remain high in females [43]. Progesterone is a gonadal hormone synthesized in large proportion by the ovary in females and, in lesser amounts, by the testicles and adrenal cortex in rats [44]. Progesterone has been shown to exert significant protective effects in a variety of experimental model factors that mimic brain dysfunction seen with old age or neurodegenerative diseases related to age, such as in Alzheimer's disease [44]. Furthermore, it has been shown that progesterone decreases neuronal death resulting from global ischemic episode [45]; it may induce a remyelination of nerve fibers [46], and it increases hippocampal synapses [47].

The calcium-binding protein (CaBP) CB is shown as an excellent marker of SCN cells in marmosets [10, 11]. CB is part of a group of CaBPs termed EF-hand of low molecular weight, and it is associated with the reduction and the control of cytoplasmic Ca^2+^ concentration, therefore being called “buffering” [48]. In our study, we found a significant decrease in the number of CB-IR cells in the SCN of aged marmosets compared to adult animals. This reduction of CB-IR neurons may be related to circadian behavioral changes seen in some aged animals, such as the rhythm of locomotor activity in rodents [49] and primates [50]. Moreover, studies have shown that the decrease of intracellular CB may result in increased vulnerability to degeneration of central and peripheral neurons [51, 52].

The labeling pattern of CB in the SCN cells of adult marmosets is characterized by nucleus almost completely stained [53], thus resembling the pattern found in hamsters and* Arvicanthis* [54]. However, in lemurs (*Microcebus murinus*), a nonhuman primate also, there is found a CB marking mainly concentrated in the central region of the nucleus [53]. In aged marmosets, the labeling pattern of CB in the SCN cells keeps appearing (i.e., nucleus almost completely stained); however, a substantial reduction of CB-IR neurons was evident. Areas without cells were more frequently found in the SCN of aged marmosets. The reduction of CB-IR neurons observed in aged animals was quite similar to what is seen in sections of aged SCN to NeuN-IR and histological Nissl staining. Thus, these findings contribute to hypothesis that neurodegeneration is occurring in the SCN of aged marmosets.

The ability of CB binds to the Ca^2+^ seems to confer protection against agents that promote cell death [55]; for example, neuronal CB-IR in the hippocampus has been shown to be resistant to induced neurodegeneration by a hiperactivation of excitatory amino acids, as well as by hypoxia/ischemia, both greatly increasing intracellular Ca^2+^ levels [56]. Therefore, the decrease of CB-IR in the SCN in aged marmosets may contribute to the reduction of cells observed with the use of other staining techniques, such as Nissl and NeuN.

Wu et al. [55] in a study using senior marmosets found a substantial reduction in regional CB-IR neurons in the cholinergic basal forebrain (BFCN). In this study, authors classified animals as seniors when they were six years old. However, according to Geula et al. [57], the appearance of senile plaques in the cerebral cortex of marmosets starts to occur at seven years of age. It is important to mention that our animals were judged aged nine years old, while at six years old, they were considered adults. However, when we analyzed our correlation data, we found a marmoset of 6 years and 3 months of age (the eldest adult), which shows discrepant cells reduction compared to other adult animals, thus resembling the morphological aspects of aged marmosets. This pattern was evident in all marks used, such as CB-IR, NeuN-IR, and also Nissl-stained cells. Two recently published studies, Tardif et al. [42] and Ross et al. [58], actually showed aspects of senescence in animals from six years of age.

Lesions of CB-IR cells in the SCN lead to a failure in the circadian rhythm regulation of body temperature, heart rate, melatonin, and cortisol, suggesting that CB is required to support circadian rhythm functioning [59]. In a study with hamster, a partial lesion restricted to the central portion of the SCN produces a decrease in the circadian rhythm activity. Interestingly, in hamsters this central portion of SCN is characterized by a rich subnucleus of CB-IR neurons. Moreover, transplantation of CB-positive cells in the injured area restored rhythm, indicating that this subregion is essential for the maintenance of locomotor activity [59] and thus may result in large changes in locomotor activity seen in aged animals [49]. Although the study of the circadian rhythm pattern of aged marmosets is still being developed, we believe that the marmoset presents the same behavior activity alterations seen in other primates [50, 53] and rodents [20, 49].

We suggest that the loss associated with the CB-IR neurons in SCN of aged marmosets may increase the vulnerability of these neurons to degenerative processes that occur in aging, such as those involving increased intracellular Ca^2+^, favoring neuronal death.

ROD analysis showed a significative increase of GFAP expression in SCN of aged marmosets compared to adults. The increase of glial expression in response to neuronal decrease is a recognized pathophysiological reaction, which is mainly seen in neurodegenerative diseases, such as Alzheimer's disease and other neurological disorders [60].

Measurements of gliogenesis rate have been used to estimate the regional overproduction of glia in response to regional decrease of neurons in a variety of disease conditions, as well as during normal aging [61, 62]. It has been suggested that the increased expression of GFAP during aging occurs due to an increase in protein damage due to oxidative processes, in all biological tissues, including the nervous system [61]. In this study, we observed a gliogenesis in response to neuronal decline in aged SCN, different from what is shown in rat and rhesus monkey, in which no changes in glial expression were found [25, 26]. Some studies reported that aged female mice showed an increase of approximately 20% of GFAP (protein) and 25% GFAP mRNA in hypothalamus and thalamus [63] and hippocampus [64] in response to neurodegeneration. Therefore, it is possible that the same effect might occur in our older animals, including in the SCN, supporting the idea that there might be a compensatory process between neuronal death (neurodegeneration) and gliogenesis in the aged marmosets SCN.

## 5. Conclusions

A morphometric analysis of SCN in aged female marmosets compared with adult animals showed a significant decrease in the number of neurons stained with Nissl and labeled immunohistochemically for NeuN, a specific marker for neurons. Other two protein markers, CB which is used as an excellent marker for SCN cells in marmoset, and GFAP were observed. This neuronal decline in aged animals may be responsible for changes seen in the pattern of expression of biological rhythms in animals during the aging process.

## Figures and Tables

**Table 1:** Summary of subjects used in the present study.

Animal	Age (years)	Weight (g)
Marmoset 2	6.3	321
Marmoset cnq6	4.0	302
Marmoset s.15	4.9	299
Marmoset s.19	3.6	260
Aged marmoset 3	11.8	357
Aged marmoset 4	12.11	322
Aged marmoset 5	9.11	367
Aged marmoset 6	11.6	355
